# Automated system utilizing non-invasive technique mammograms for breast cancer detection

**DOI:** 10.1186/s12880-024-01363-9

**Published:** 2024-08-07

**Authors:** Hazem M. Ammar, Ashraf F. Tammam, Ibrahim M. Selim, Mohamed Eassa

**Affiliations:** 1 Computer Science Department, Thebes Higher Institute for Engineering, Cairo, Egypt; 2 Computer Engineering Department, Faculty of Engineering and Technology, Arab Academy for Science, Technology & Maritime Transport (AASTMT), Cairo, Egypt; 3 Faculty of Computers and Artificial Intelligence, Sadat City University, Sadat City, Egypt; 4 Computer Science Department, Faculty of Information Systems and Computer Science, October 6 University, Giza, Egypt

**Keywords:** Breast cancer, Decision tree, Supervised learning algorithms, Classification, Random forest

## Abstract

In order to increase the likelihood of obtaining treatment and achieving a complete recovery, early illness identification and diagnosis are crucial. Artificial intelligence is helpful with this process by allowing us to rapidly start the necessary protocol for treatment in the early stages of disease development. Artificial intelligence is a major contributor to the improvement of medical treatment for patients. In order to prevent and foresee this problem on the individual, family, and generational levels, Monitoring the patient's therapy and recovery is crucial. This study's objective is to outline a non-invasive method for using mammograms to detect breast abnormalities, classify breast disorders, and identify cancerous or benign tumor tissue in the breast. We used classification models on a dataset that has been pre-processed so that the number of samples is balanced, unlike previous work on the same dataset. Identifying cancerous or benign breast tissue requires the use of supervised learning techniques and algorithms, such as random forest (RF) and decision tree (DT) classifiers, to examine up to thirty features, such as breast size, mass, diameter, circumference, and the nature of the tumor (solid or cystic). To ascertain if the tissue is malignant or benign, the examination's findings are employed. These features are mostly what determines how effectively anything may be categorized. The DT classifier was able to get a score of 95.32%, while the RF satisfied a far higher 98.83 percent.

## Introduction

Cancer of the breast occurs when cells in the breast spread uncontrollably manner. Cancer of the breast has several different forms, depending on which breast cell type transforms into cancerous. It's important to remember that breast cancer can start anywhere in the breast. Lobules, ducts, and connective tissue make up the breast. The lobules contain the milk-secreting glands. Tubes called ducts transport milk from the mammary glands to the nipple. Connective tissue is made up of both fibrous and fatty tissue, and it's responsible for keeping everything together. In most cases, the ducts or lobules are the starting point for breast cancer. Through the lymphatic and circulatory systems, Breast cancer has the potential to spread to other parts of the body or become uncontrollable. Breast cancer "metastasized" significantly, which indicates that it spread to different bodily parts [1]. The two most frequently detected kinds of breast cancer are invasive ductal carcinoma and invasive lobular carcinoma. When invasive ductal carcinoma affects the breast ducts, the cancer cells first develop inside the ducts before developing outside of them and disseminating to other areas of the breast tissue. Aggressive cancer cells are more prone to metastasis or spread to different parts of the body. During the invasive lobular carcinoma process, cancer cells originate in the lobules and then spread from the lobules to the breast tissues that are close to one another in location. These cancer cells can potentially invade other sections of the body and spread throughout the body [2]. There were about 2.3 million new cases of breast cancer detected in women worldwide in 2020, with an estimated 685 thousand fatalities. The highest incidence was recorded in Belgium (112.3 per 100,000 people), while the lowest was recorded in Iran (35.8 per 100,000 people), and Fiji had the highest rate in fatality (41.0 per 100,000), while South Korea had the lowest rate (6.4 per 100,000) [3].

In recent years, among women's major causes of mortality, breast cancer has recently become more prevalent. Mammography screening programs aim to catch cancer early, which could save lives but also result in unnecessary diagnoses and treatments for many women. Because slow-growing tumors are more likely to be found during screening (length bias), the risks associated with the needless treatment of over-diagnosed tumors have the potential to offset or minimize the possible benefits of the screening [4]. Many methods improve the resolution of these images for a better diagnosis [5, 6].

In various fields, including medicine, artificial intelligence (AI) is being used to promote, assist and solve problems, as some images are of low quality and contain noise, making it difficult for medical professionals to diagnose breast cancer in them. In order to diagnose patients quickly and accurately, we are turning to contemporary artificial intelligence technologies [7, 8]. By using AI-based methodologies, it is possible to increase the accuracy of digital mammography imaging, which includes DT, Naive Bayes (NB), Support Vector Machine (SVM), K-Nearest Neighbor (KNN), and to create software that uses mammography and scripts to look for breast cancer [9–12].

For training classification models using data, many supervised learning techniques have been put out in the literature. These methods are still not very accurate in identifying benign from malignant tumors, and they are also not very accurate in diagnosing breast cancer. In the classification problem, where the distribution of classes is not uniform, datasets are also unbalanced. In this paper, using statistical data from mammograms, we plan to build a model that can distinguish and categorize breast cancer as malignant or benign tumors, as well as predict its future occurrences depending on some other checkups, including pathological history or genetic factors. Therefore, the main contributions are as follows. We developed a classification model through RF and DT based on a series of mammograms that were examined to clarify breast cancer and the distinctions between malignant and benign cancers, whether radiotherapy, chemotherapy, or surgery. Also, recall, Precision, and f1-score are among the measures that were used to evaluate imbalanced datasets to gain more knowledge.

The remaining sections of the paper are presented as follows: related works are included in Related works section. The proposed system and dataset are described in Methodology sections and Discussion and Results section, while Experimental results section demonstrates the experimental results. In the final section, Conclusions section, conclusions are described.

## Related works

This section will explore some of the earlier research that was conducted to identify breast cancer using machine-learning approaches. These researchers have employed a wide range of approaches.

Vishal Chauhan et al. [13] This survey study offers an overview of the various machine-learning methods for breast cancer detection. Furthermore, offers a comparative evaluation of various machine-learning methods for the identification of breast cancer.

Three different machine learning techniques— Bayesian Networks (BN), RF, and SVM — were looked at in comparative research on this topic by Dana Bazazeh and Raed Shubear [14]. They used the original Wisconsin breast cancer dataset as a training set of data. Results from the simulations show that the classification performance changes depending on the selected method. The findings show that SVM performs optimally in terms of precision, specificity, and precision. On the other hand, RF offers the best potential for correctly identifying malignancies.

Using the morphological properties of breast ultrasonography, Zeebaree et al. [15] built a CAD that uses ML and segmentation for increasing regions. The approach extracts feature from the ROI using a hybrid model. In place of a single feature, we have included 7 moments, FD, and HOG. There were a total of 250 ultrasound pictures utilized, 100 of which showed benign lesions and 150 showing malignant ones. Ultrasound images may be accurately classified with a success rate of 93.1% for cancerous and 90.4% for benign using the ANN.

Jalalian, et al. [16] This article explains why it's so important to detect cancer early so it may be treated successfully. So, Computer-Aided Detection, or CAD, is a method that is essential for spotting breast cancer at an early stage. This study describes the many irregularities that could be breast cancer and how to spot them using computer-aided diagnosis (CAD) methods. Abnormalities such as mass detection, abnormality classification, structural distortion, and bilateral asymmetry are discussed.

Wei et al. demonstrated a technique for automatically classifying breast cancer from breast imaging data. [17]. The proposed method uses the textural and morphological properties of tumor images to categorize them as benign or malignant. Totaling 1061 ultrasound images, the proposed approach depicts 589 malignant and 472 benign tumors. A few features that were extracted from the region of interest (ROI) include compactness, elliptical direct least-squares fitting, and radial range spectrum. We classified morphological traits using the SVM classifier. In light of the results, the accuracy rate was estimated to be 74.94%, the sensitivity rate was determined to be 66.37%, the specificity rate was revealed to be 86.87%, and the precision rate was reported to be 85.23%.

Liu et al. have proposed a computer-aided design (CAD) system for categorizing breast tumors based on the extraction of edge features where Computer-aided Design for diagnostic methods (CAD) helps radiologists improve the interpretation of mammograms to detect breast cancer. [18]. Several morphological metrics, such as regularity, aspect ratio, roundness, elasticity, and roughness, were calculated from the ROI. They also used roundness, another extracted attribute, to help them assess whether the lesions were malignant or benign. The proposed method included 192 ultrasound examinations in total, of which there 71 were benign and 121 were malignant. With the proposed technique, they were able to reach a sensitivity of 47.62%, an accuracy of 67.31%, a Negative Predictive Value (NPV) of 69.44%, a specificity of 80.65%, and a Positive Predictive Value (PPV) of 62.50%.

A Hough transform was proposed by R. Vijayarajeswari et al. [19] as a technique for determining mammography picture properties. The SVM classifier used takes these properties as inputs. The SVM classifier's accuracy range, which is greater than the accuracy range of the linear discriminant analysis (LDA) classifier, was 94%. (86 percent).

Rakesh Kumar et al. [20] This study compares Gradient Boosting with Light Gradient Boosting (LightGBM) which is utilized for classification, ranking, and other machine learning applications and is based on decision tree algorithms, with trials conducted using a labeled dataset of breast cancer. Compared to the Extreme Gradient Boosting (XGBoost) technique which is a bagging-based boosting approach that trains several decision trees and then combines the output, the LightGBM approach has been found to be less accurate.

M. Karaiyarasi et al. [21] performed the standard classification techniques SVM, ANN, and logistic regression. The breast cancer dataset from Kaggle is utilized. The test and training data were divided by 7:3. Important features are determined by the correlation matrix. Metrics found the most effective classification models after creating the models. The results of future optimization techniques will be above 99% in substantial numbers.

In the study by A. O. Ibrahim et al. [22], A radial basis function network (RBF)-based CAD system for breast diagnostics has been proposed. The procedure of classifying lesions with RBF network classifiers makes use of the decision-making system. The procedure of classifying tumors with RBF network classifiers makes use of the decision-making system. This study aims to investigate the link between multilayer perceptron (MLP) algorithms and RBF neural networks. Overall, the RBF neural network performed better than the MLP method, with an accuracy of 79.166 percent vs. 54.1667 percent. These results confirmed the superior classification accuracy of the RBF neural network when applied to mammography pictures.

In order to classify breast cancer cases in the cloud, Lahoura et al. [23] proposed an extreme learning machine (ELM)-based machine learning system. The ELM model was implemented following the use of Naive Bayesian, SVM, AdaBoost, k -NN, and perceptron. Information from the Wisconsin Breast Cancer Data (WBCD) registry was retrieved. There were 569 records in the database and 32 corresponding characteristics. According to the data, the approach has a 98.68% success rate, a 91.30% recall rate, a 90.54% precision rate, and an 81.29% F1-score.

## Methodology

Build models that can recognize and categorize breast cancers as benign or malignant using data from mammograms and other studies as well as statistical features that anticipate future (long-term) occurrence. Usually, the patient goes to the examination room for the examination, and the assistant helps the patient to obtain the X-rays and necessary data in mammography. The system uses this image as input for extracting some of the key features. These characteristics allow the model to categorize and separate image categories to distinguish between benign and malignant breast tissue, track patient recovery and treatment, and determine whether a patient is at the individual or family level. You can predict whether generations will emerge in the long run. When a benign tumor is found, the medical facility system is alerted, and the patient is sent there to be evaluated for cancer diagnosis, non-cancerous status monitoring, and any necessary treatment. Finally, once cancer has been diagnosed, the patient will be admitted to the hospital for the needed treatment after the cancer stage has been determined and the hospital system has been informed. Additionally, it makes predictions about future cancer recurrence in patients and future cancer in the next generation.

### Proposed system

**Fig. 1:**
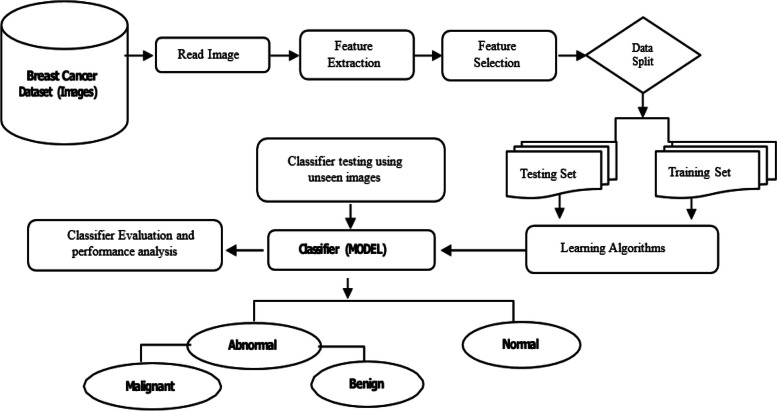
System for detecting breast cancer in its general form, while Breast cancer types are described and categorized using a classifier model for breast cancer prediction. The model that is proposed is based on feature extraction from a set of diverse images with identified category labels and feature selection to identify the most crucial and target-related attributes. Based on image features, breast cancer is separated into benign and malignant categories using the classifier learning method. The proposed system consists of several phases

Breast cancer types are described and categorized using a classifier model for breast cancer prediction. The model that is proposed is based on feature extraction from a set of diverse images with identified category labels and feature selection to identify the most crucial and target-related attributes. Based on image features, breast cancer is separated into benign and malignant categories using the classifier learning method. The proposed system consists of several phases, as shown in Fig. 1, Taking features out of a picture is the most important feature. The characteristics that are most crucial for the target class are chosen using the data gain technique, which is used to evaluate the relationship between features and labels. Equation (1) calculates the gain between the i^th^ feature fi and the labels. Equation (3) determines the expected data required to classify a tuple in D, while equation (2) determines the anticipated data required [24].

1 $$\text{Gain }\left(\text{f}\right) =\text{ Info }\left(\text{D}\right) - {\text{Info}}_{\text{f }}\left(\text{D}\right)$$

2 $$\text{Info }\left(\text{D}\right) = -\sum\nolimits_{\text{i}=1}^{\textrm{n}}{\text{P}}_{\textrm{i}}{\text{log}}_{2}\left({\text{P}}_{\textrm{i}}\right)$$

3 $${\text{Info}}_{\text{f }}\left(\text{D}\right) = \sum\nolimits_{\text{i}=1}^{\text{n}}\frac{\left|\text{Dj}\right|}{\left|\textrm{D}\right|}\text{ X Info }(\text{Dj})$$

### Dataset

**Fig. 2:**
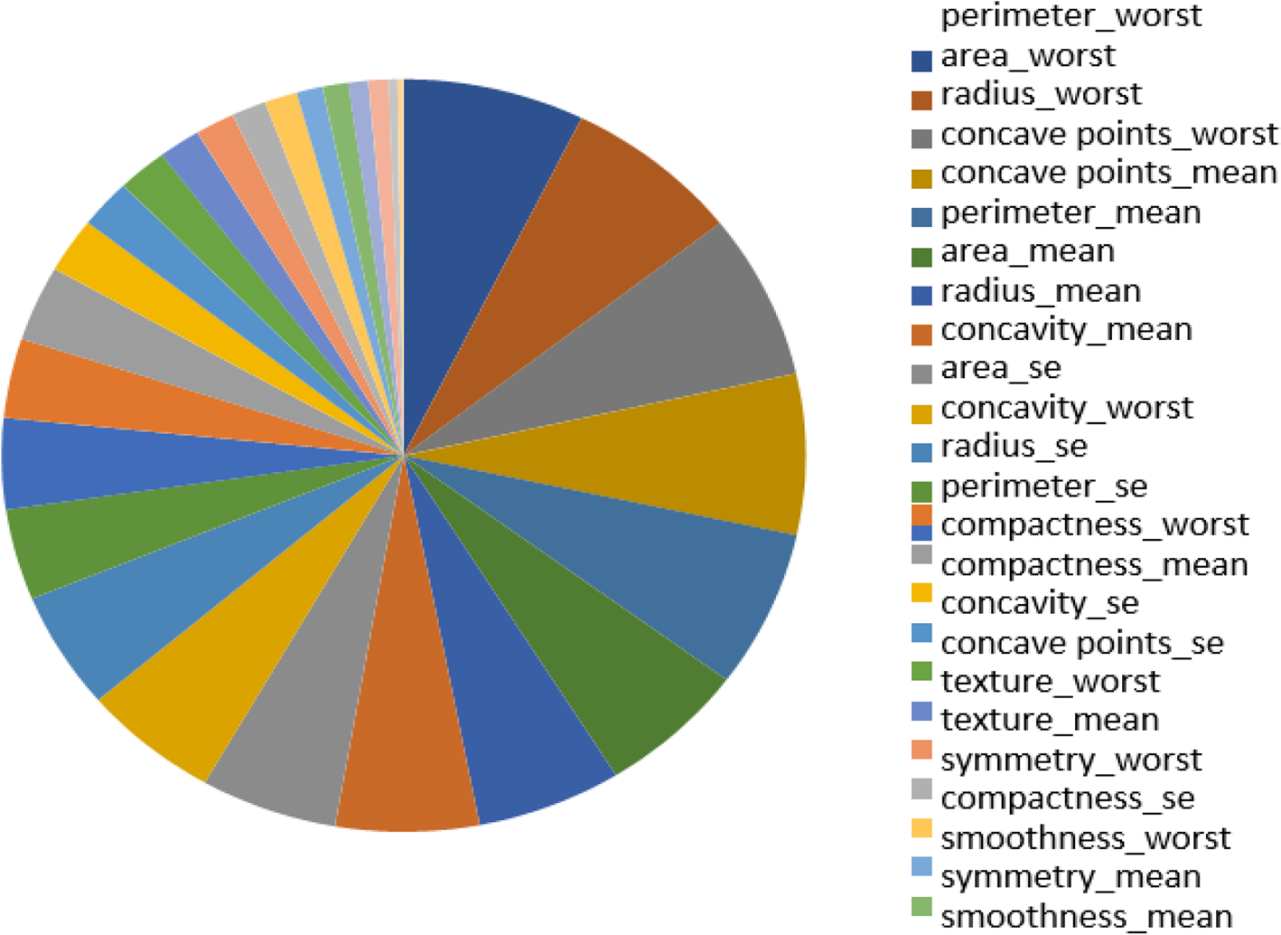
Feature ranking according to its weight while the features that were utilized as a data set to train a classifier to evaluate whether breast tissue is cancerous or not

The most frequent type of cancer among women is breast cancer. Features are calculated from digitalized pictures of breast masses obtained through fine-needle aspiration (FNA). They serve as a representation of the characteristics of the image's cell nuclei. The Breast Cancer Wisconsin Dataset (Diagnosis) was used for the study's dataset. Figure 2 displays the features that were utilized as a data set to train a classifier to evaluate whether breast tissue is cancerous or not. With 570 samples in it. There are 30 features per sample [25].

**Fig. 3:**
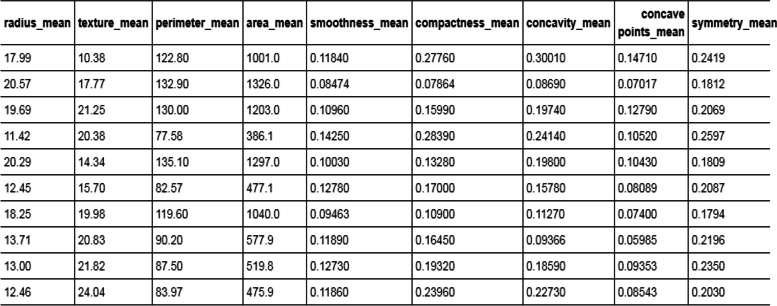
Sample of breast cancer dataset which split into testing and training groups. 570 samples are included in the data set

Using a data split, the dataset is split into testing and training groups. 570 samples are included in the data set, as shown in Fig. 3. The 30 main characteristics of each sample include average symmetry, worst perimeter, average compactness, worst concave, and so on. In data splitting, the dataset is split at random into 30% for model testing and 70% for model training [25], as shown in Fig. 3. The dataset assumes that all features extracted from images are statistical features, so they are more visible and unaltered than features extracted from molecular classification techniques.

For classification issues where the distribution of classes is not uniform, imbalanced datasets are a specific instance. Because it can be quite deceptive, accuracy is not the best metric to employ when assessing imbalanced datasets. Precision, recall, and f1-score are metrics that can offer deeper insight.

## Discussion and results

This section presents the proposed system's actual performance evaluation as well as a discussion of that evaluation. The effectiveness of the system that is being presented is evaluated based on the findings of many different kinds of experiments.

### Performance metrics

**Fig. 4:**
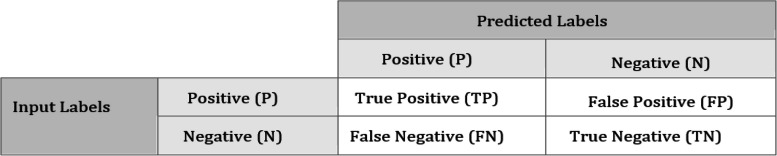
Matrix of confusion between the labels of positive and negative which demonstrates the various measures used to assess the model's accuracy. These metrics define the, false positive (FP), true positive (TP), false negative (FN), and true negative (TN)

Evaluation and testing at the end of the stage for classifiers to get to the right conclusion about the experiment's overall results is necessary to evaluate and evaluate the classifiers to corroborate the findings of the experiment. Additionally, the classifier's capacity to differentiate between the several picture classes must be evaluated. The accuracy of the model is evaluated utilizing a range of measures, such as F-measure, precision, and recall, as shown in Fig. 4 [24], which demonstrates the various measures used to assess the model's accuracy. These metrics define the false positive (FP), true positive (TP), false negative (FN), and true negative (TN).

As can be seen in Fig. 4, a confusion matrix may be used to visualize and evaluate a classifier's performance. As the name implies, True positive (TP) counts the number of people who are correctly categorized as belonging to the ill positive class. The true negative (TN) is the proportion of healthy individuals who are correctly classified as belonging to the negative class. False positives (FP) are the number of times healthy persons have been misdiagnosed as ill. When the estimated number of healthy people is wrong, a false negative (FN) results. You may use a confusion matrix to figure out your recall, precision, accuracy, and F-measure.

Precision: utilized to evaluate the classifier's accuracy, and its value can be derived based on the given information This can be demonstrated by looking at equation 4, which demonstrates how the precision measure, by comparing the observed TP to those predicted, reveals how accurate the proposed procedure's behavior.

4 $$\text{Precision }= \frac{{\text{T}}_{\text{p}}}{{\textrm{T}}_{\textrm{p}} + {\textrm{F}}_{\textrm{p}}}$$

Recall: recall is a measurement that determines how complete the classifier findings are. In Equation 5, recall or sensitivity is defined as the proportion of correctly detected positive samples. It is calculated by using that equation.

5 $$\text{Recall }= \frac{{\text{T}}_{\text{P}}}{{\textrm{T}}_{\textrm{N }}+{\textrm{ F}}_{\textrm{N}}}$$

F-measure is a harmonic average that equally weighs recall and precision. It allows a model's performance to be described and compared using a single score that takes precision and recall into consideration and may be determined using Equation 6.

6 $$\text{F}-\text{ measure }= 2 * \frac{(\text{ precision }*\text{ recall })}{(\textrm{ precision }+\textrm{ recall })}$$

The accuracy of these checks, which are designed to establish what percentage of samples have been correctly classified. Equation 7 evaluates the extent to which the outcomes are consistent with the outcome that was originally calculated.

7 $$\text{Accuracy }= \frac{{\text{T}}_{\text{P}} +{\text{ T}}_{\text{N}}}{{\textrm{T}}_{\textrm{P }}+{\textrm{ T}}_{\textrm{N }}+ {\textrm{F}}_{\textrm{P }}+{\textrm{ F}}_{\textrm{N}}}$$

## Experimental results

**Fig. 5:**
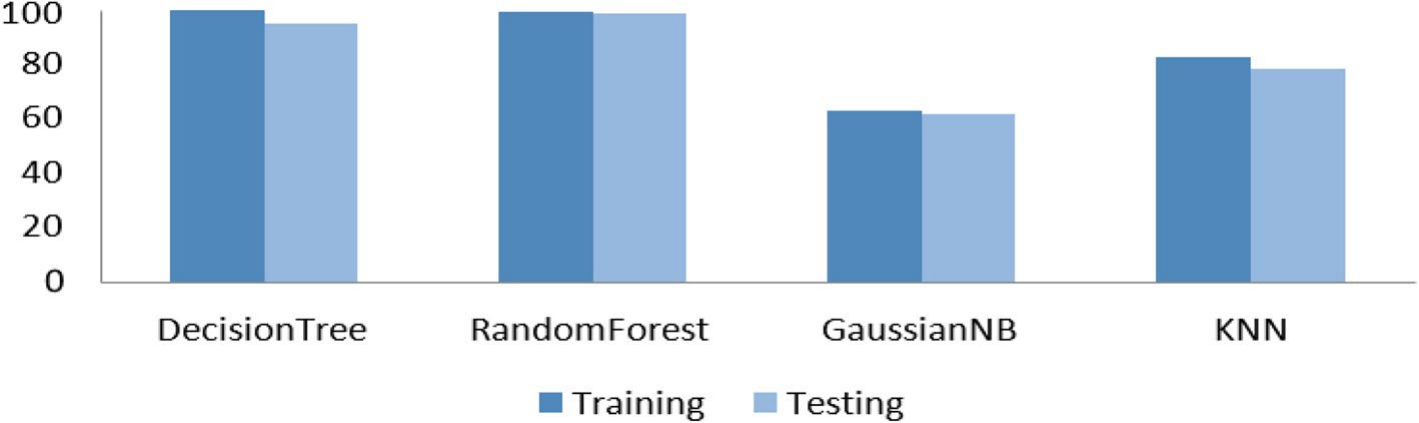
Overall accuracy for 70:30 data split for each classifier which achieve a high level of accuracy, and forecast the kind of breast cancer that is present based on mammography

We have explained in detail the experimental results acquired. Our focus is on the accuracy of disease prediction by the various existing approaches. Table 1 presents the gained results with the data split ratio as 70:30. It is important to look first at the accuracy of the used algorithms. As can be seen in Table 1, the accuracy in the cases of KNN, Gaussian Naive Baise (NB) Classifier, DT classifier, and RF classifier are 78.36, 61.98, 95.32, and 98.83, respectively. These results are clarified in Figure 5, where the accuracy of all algorithms is depicted for clarity. This indicates that the proposed model overcomes the reset of the algorithms in terms of accuracy. To train classification models from data, a variety of algorithms are utilized. These models can subsequently be utilized to forecast the classes of novel datasets that include previously unseen samples. As can be seen in Fig. 5, we put the majority of these classification algorithms through the data split technique to find the optimal classifier, achieve a high level of accuracy, and forecast the kind of breast cancer that is present based on mammography. Following the results of the experiments, we reported the two classifiers that we deemed to be the most effective overall, specifically the DT and RF classifiers. Both of these classifiers have the highest accuracy. There are two steps involved in the learning process. The first step is known as "learning," and it involves building a model with the help of the training data. The second step, known as "testing," involves evaluating the correctness of the model with previously unseen test data.

**Table 1:** Overall accuracy for each classifier with a 70:30 data split which presents the gained results with the data split ratio as 70:30

Methods	Training	Testing
Decision Tree	100.00	95.32
Random Forest	99.74	98.83
Gaussian NB	62.81	61.98
KNN	82.91	78.36

**Fig. 6:**
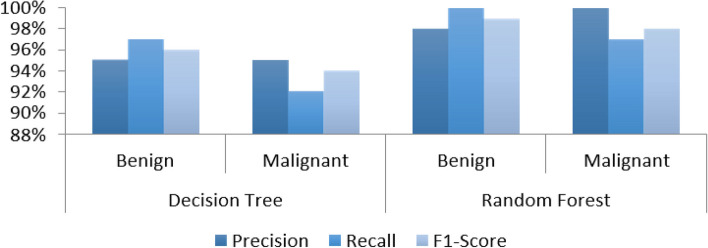
F1-score, recall, and precision for 70:30 data split for each classifier which demonstrates the overall accuracy for the classifiers

The overall accuracy is shown in Fig. 6 and Table 2 using a 70:30 data split for RF and DT only. Python software has analyzed and verified the outcomes of the experiments. Python has been used to implement the experimental findings and their implications.

**Table 2:** Results with a 70:30 data split A Python software has analyzed and verified the outcomes of the experiments. Python has been used to implement the experimental findings and their implications

Model	Target	Precision	Recall	F1-Score	Support	Accuracy
Decision Tree	Benign	95%	97%	96%	106	95.32%
	Malignant	95%	92%	94%	65	
Random Forest	Benign	98%	100%	99%	106	98.83%
	Malignant	100%	97%	98%	65	

The confusion matrix is a summary and visual depiction of a table used to assess the effectiveness of a categorization system. According to Equation 4, the number of properly identified samples is displayed in Table 3, which depicts a confusion matrix in which healthy tissue is referred to as benign and malignant tissue is referred to as cancerous.

**Table 3:** utilizing the PS (70–30) method, the RF and DT confusion matrix which depicts a confusion matrix in which healthy tissue is referred to as benign and malignant tissue is referred to as cancerous

Random Forest (RF)				Decision Tree (DT)			
Training		Testing		Training		Testing	
251	0	106	0	251	0	103	3
1	146	2	63	0	147	5	60

### Comparison with previous work

The current model shows strong competitiveness over previously related work with higher success results than most related work. Table 4 shows a comparison of the accuracy of the current work classification as compared to the results of the related works.

**Table 4:** Comparison of the results obtained by the proposed model and other previous models which presents an accuracy comparison between the proposed model and the other models

Methods	Accuracy	No. of samples
Zeebaree et al. [15]	93.1%	250
Wei et al. [17]	74.94%	1061
Lahoura et al. [23]	98.68	569
Proposed model (Decision Tree)	95.32%	570
Proposed model (Random Forest)	98.83%	570

## Conclusions

Using machine learning and artificial intelligence to construct classifier models that aid physicians in making definitive diagnoses and predicting or detecting illnesses at an early stage is the most promising area of development in medical applications such as diagnosis and therapy. Get a proper diagnosis and then treat it. RF-DT was utilized for categorizing data in this investigation. Mammograms may identify both cancerous and noncancerous growths in the breast. The maximum accuracy, 98.83 percent, was achieved by the RF classifier, followed closely by the DT classifier, at 95.32 percent. The most salient characteristics of a data collection are isolated using RF classifiers. Tenfold was the model's intermediate phase. At each iteration, we resample the data and train the classifier using a new fold. Results from the sample models that were not shown were therefore reliable. The results of this research demonstrated the CAD tool's potential by surpassing prior classification methods. The establishment of the instrument may aid less experienced oncologists in providing curative drugs. Future work will employ deep learning techniques and involve the creation of new datasets in addition to the utilization of existing datasets like MIAS, INbreast, and CBIS-DDSM.

## Data Availability

The datasets generated and/or analyzed during the current study are available in the (breast cancer Wisconsin (diagnostic) dataset] repository, (https://archive.ics.uci.edu/ml/datasets/breast+cancer). All data generated or analyzed during this study are included in this published article (https://docs.google.com/document/d/1m_Idnr11fwWmIwVEY8uU3P7FwvZqW5Hm/edit?usp=drivesdk&ouid=117190551595426872884&rtpof=true&sd=true).
